# Local Treatment of Diphtheria

**Published:** 1896-11

**Authors:** 


### Local Treatment of Diphtheria.

The following is extracted from a paper by Dowling Benjamin, Camden, N. J., in the *Journal American Medical Association*, October 17, 1896:

If it be true (and I do not think that it can be successfully disputed now, that diphtheria is due to the presence of germs in the pharynx), why would not germicides destroy them? For we know that we possess a number of antiseptic or germicidal medicines that are invariably fatal to germs, and can be used in quantities sufficient for that purpose without any detriment to the patient. Holding the view of the disease that I have advanced, I could never understand why a local treatment would not be all that was required.

Professor Loeffler, after various experiments, suggested a combination of:

| | |
|---|---|
| Menthal cryst | 10 gm. |
| Touluol q. s. ad fac | 36 c.c. |
| Alcohol absolute | 60 c.c. |
| Liquor ferri sesquichloridi | 4 c.c |

I have found the formula of Professor Loeffler to be disagreeable to the patient, while the one I use has not that objection and is quite as efficient.

| | |
|---|---|
| Acid. acet. dilut | fl.ʒii |
| Pot chlorat | ʒss |
| Acid. carbol | gtt.i |
| Tinct. ferri chloridi | gtt.v |
| Pulv. Alumen | gr.v |
| Acid. salicylic | gr.i |
| Glycerin | fl.ʒss |
| Aqua ros | fl.℥ss |
| Aqua q. s. ad | fl.℥iv |

Misce. Sig. Use as directed.

It is a clear, permanent liquid of a purple color.

In making application to the throat of diphtheretic patients the doctor or nurse may use a disc of glass held between his face and the patient, to prevent infection from the sudden coughing and spitting of the patient.

I always give tincture ferri chloridi in large and frequent doses, and I believe it produces favorable conditions of the blood, as it does in many other cases of septicemia, notably erysipelas, which often disappears under this treatment alone.

One of the benefits derived from this remedy is its local action while being swallowed.

Result under the local antiseptic treatment: 100 cases, 100 recoveries."

[He "could never understand why a local treatment would not be all that was required;" yet gives tincture ferri chloridi in large and frequent doses, "believing that it produces favorable conditions of the blood." Humph!—Ed.]

